# Casting the net broader to confirm our imaginations: the long road to treating wasting disorders

**DOI:** 10.1002/jcsm.12256

**Published:** 2017-11-23

**Authors:** Stephan von Haehling

**Affiliations:** ^1^ Department of Cardiology and Pneumology University of Göttingen Medical Center Göttingen Germany

**Keywords:** Cachexia, Sarcopenia, Wasting, Heart failure, COPD, Cancer

## Abstract

Wasting embraces muscle and tissue wasting in sarcopenia and cachexia. This article describes recent advances in the field published in the Journal of Cachexia, Sarcopenia and Muscle concerning diagnostic tools, biomarker development, pathophysiology, and treatment. Studies discussed herein embrace those on sarcopenia and cachexia in heart failure, chronic obstructive pulmonary disease, and cancer including also animal models.

## Introduction

Wasting is an umbrella term that describes loss of lean and non‐lean tissues as well as loss in bone mineral density. Wasting thus embraces both sarcopenia and cachexia. From a clinical standpoint, sarcopenia may be divided into primary sarcopenia as part of the ageing process and into secondary sarcopenia that develops in the context of chronic illness. The term describes the loss skeletal muscle mass, which is not usually associated with weight loss but with loss of functioning muscle tissue and strength.^1^ Cachexia, on the other hand, develops as a consequence of a chronic inflammatory disease such as chronic heart failure, chronic kidney disease, chronic obstructive pulmonary disease (COPD), rheumatoid arthritis, or cancer. The determining feature of cachexia is the loss of body weight, which can be the result of loss in fat tissue, skeletal muscle, or both. Whenever such wasting is present in the context of chronic disease, this can usually be regarded as a *signum mali ominis*. Frailty, on the other hand, is a functional term that describes a functional result of muscle loss and cachexia leading to dependency, increased morbidity, and mortality, all of which are the result of functional decline leading, for example, to falls.^2^

The aim of the following overview is to provide insight into recent developments in the fields of body wasting and frailty with a focus on data published in the *Journal of Cachexia, Sarcopenia and Muscle*. The journal, founded in 2010, is focused on the dissemination of knowledge to both clinicians and researchers with a primary aim of highlighting data in the field of wasting across all the life sciences. Another important medium for dissemination is the now annual Cachexia Conference that was last held in Paris in 2015^3^ and in Berlin in 2016, after which the Cachexia Conference went annual.^4^ Being such a niche journal, the journal now receives an increasing number of submissions, downloads as well as an increasing impact factor.^5^, ^6^ In this sense, the American architect, designer, and writer Richard Buckminster Fuller (1895–1983) was right when he wrote that ‘You never change things by fighting the existing reality. To change something, build a new model that makes the existing model obsolete’.

## Sarcopenia

### Prevalence, screening, and diagnosis

**Figure 1:**
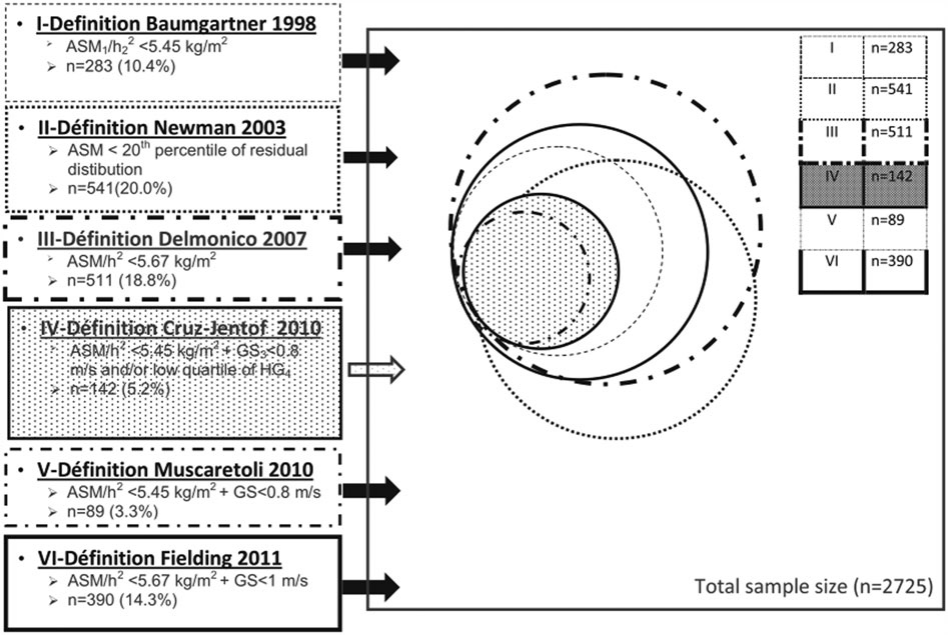
Distribution of a participant identified as sarcopenic according to the different definitions. ASM, appendicular skeletal muscle mass; h, height; GS, gait speed; HG, handgrip strength. Reproduced from Dupuy *et al*. 14

The diagnosis of sarcopenia remains rare outside the field of geriatrics, and therefore, the introduction of an ICD code for sarcopenia represents a major step forward in recognizing sarcopenia as a disease.^7^ Even though a number of bodies have recommended diagnostic criteria for sarcopenia, screening, use of diagnostic tools, and treatment remain underutilized.^8^ The heterogeneity of definitions proposed for the diagnosis of sarcopenia makes matters rather worse.^9^, ^10^, ^11^, ^12^, ^13^ Naturally, this fact is most important in the elderly. Using data from 3025 non‐disabled women aged 75 or older, the cross‐sectional EPIDOS study by Dupuy *et al*.^14^ showed the prevalence of sarcopenia ranging from 3.3% to 20.0% according to which one of six definitions was used (*Figure* 1). In this context, it was interesting to note that only 85 participants (3.1%) were identified to have sarcopenia according to all definitions.^14^ This study underscores the importance of having unified criteria that identify a clinically relevant cohort of affected subjects. Before such unifying definition is available and to better understand patients at risk of or with manifest sarcopenia, rapid screening remains of utmost importance. Morley and Cao^15^ have recently noted that the different definitions all embrace muscle function and muscle mass. However, they also acknowledge that muscle quality and therefore muscle performance are not directly related to muscle mass. This point is important because sarcopenia may also be a neuromuscular junction disease, and infiltration of fat into muscle during the ageing process precludes the development of weight loss.

The easiest way of assessing sarcopenia seems to be bioelectrical impedance assessment for measuring lean mass coupled with either gait speed or handgrip dynamometry. An easy questionnaire is the SARC‐F questionnaire that is able to rapidly screen for sarcopenia, and the questionnaire's results have been found to be associated with physical performance, grip strength, and hospitalization in the previous 2 years.^16^ The SARC‐F questionnaire was studied in substudies of the African American Health Study, the Baltimore Longitudinal Study of Ageing, and the National Health and Nutrition Examination Survey. SARC‐F was evaluated here using cross‐sectional and longitudinal differences among those with high (≥4) vs. low (<4) SARC‐F scores for mortality and health outcomes. Overall, the SARC‐F questionnaire results proofed internally consistent and valid for detection of persons at risk for adverse outcomes from sarcopenia in the study cohorts investigated here.^16^ Estimates regarding the prevalence of sarcopenia using data from 18 363 people aged 65 years or above showed that sarcopenia was present 12.6% in Poland to up to 17.5% in India. The prevalence of sarcopenic obesity, a risk factor for impaired survival, for example, in cirrhosis,^17^ ranged from 1.3% in India to 11.0% in Spain. Low levels of physical activity were particularly associated with higher odds for the development of sarcopenia and sarcopenic obesity.^18^ Barbosa‐Silva *et al*.^19^ assessed the prevalence of sarcopenia as part of a cross‐sectional population‐based study in 1291 community‐dwelling elderly subjects aged 60 years or older using calf circumference measurements. Measurements were performed in the city of Pelotas in Brazil, and the authors found sarcopenia to be associated with low schooling level, being without a partner, low socio‐economic status, smoking, inactive life style, and low body mass index. The overall prevalence of sarcopenia was 13.9%.^19^ Foong *et al*. studied 636 community‐dwelling older adults whose muscle mass was measured using dual‐energy X‐ray absorptiometry (DEXA), and lower limb strength was measured via dynamometry.^20^ Not surprisingly, they found that physical activity intensity was positively associated with higher lean mass percentage and lower limb strength in a dose–response fashion. Sedentary activity was negatively associated with lean mass percentage but not with lower limb strength. This study underscores the importance of physical activity in elderly adults. In this context, handgrip strength remains one of the most importance screening tools, and a study by Leong *et al*. using data from 125 462 healthy adults from 21 countries aged 35–70 years showed that handgrip strength values differ among individuals from different geographic regions. Values were highest among subjects from Europe and North America but lowest from those from South Asia, South East Asia, and Africa and intermediate among those from China, South America, and the Middle East, buttressing the view that reference values need to adjust for ethnic backgrounds.^21^ The before mentioned data are in line with the results by Gielen *et al*.^22^ who showed that appendicular lean mass decreased from age 50 onwards, while gait speed and grip strength significantly decreased only from age 70. These authors used data from 518 men aged 40–79 years recruited for participation in the European Male Ageing Study, and they also found that the incidence of sarcopenia among their study cohort varied from 1.6% to 8.1% according to the definition of sarcopenia used. In men aged ≥70 years, low insulin‐like growth factor‐1 values were associated with a greater decrease in gait speed. However, baseline endocrine variables were not independently associated with an increased risk of incident sarcopenia by any definition.^22^ Finally, using data from a population‐based study cohort including 4425 older adults from the Third National Health and Nutrition Survey, Brown *et al*. found that the prevalence of sarcopenia was 36.5% and that sarcopenia was associated with an increased risk of all‐cause mortality in men and women; cardiovascular mortality was only increased in sarcopenic women.^23^

The problem of screening for the presence of sarcopenia, however, remains very valid, and several attempts have been made to establish a biomarker or a biomarker portfolio that could help in identifying patients with sarcopenia. C‐terminal Agrin Fragment (CAF) has been used in patients after acute stroke during early rehabilitation to identify those fulfilling criteria of sarcopenia.^24^ CAF originates from the degeneration of neuromuscular junctions, and its sub‐fragment CAF22 was elevated in patients admitted for rehabilitation after stroke. CAF22 values showed only incomplete recovery until discharge, and an association was found between CAF22 levels and parameters of physical performance, hand grip strength, and phase angle on bioelectrical impedance assessment measurement.^24^ Other markers that have been used as part of early studies include tartate‐resistant acid phosphatase 5a and epigallocatechin‐3‐gallate.^25^ However, the overall situation with regard to biomarkers remains unsatisfying, particularly concerning sensitivity and specificity.^26^ Nedergaard *et al*. studied blood samples and DEXA‐data from 41 patients with head and neck cancer as part of the Danish Head and Neck Cancer Group. They studied the collagen fragment serum markers Collagen type III propeptide (ProC3), Collagen type VI peptides containing the IC6 epitope, and Collagen type VI fragment C6M but were unable to show any correlation between biomarkers and lean body mass.^27^ It appears that these biomarkers, which worked as biomarkers of lean body mass in healthy individuals of both genders, do not work in the cancer patients investigated in this study. Because biomarkers have not emerged on the horizon in recent years, the focus remains on easily applicable muscle strength assessments such as handgrip strength and on questionnaires.

### Treatment

At least three treatment approaches suggest themselves with regard to the treatment of sarcopenia. These include nutrition, physical activity, and pharmacotherapy or potentially a combination of these three. De Vries *et al*.^28^ performed patient‐centred physical therapy as part of a randomized controlled trial that included 13 physical therapy practices with follow‐up measurements at 3 and 6 months. Eligible patients to be included in the study were aged 70 years or over and had mobility problems. The primary outcome of the study was physical activity in minutes per day. The study included 130 patients, and the between‐group difference was significant for moderate intensity physical activity in favour of the ‘Coach2Move’ group that tested the effectiveness of patient‐centred physical therapy strategy. In this group, frailty also decreased more significantly than in the usual care group, and the overall results add to an improvement in the quality‐adjusted life years. Importantly, the authors reported that their approach using physical therapy was cost‐effective.^28^ Barbat‐Artigas and colleagues^29^ studied 146 obese women with a body mass index ≥ 30 kg/m^2^ who participated in a 3‐week usual and institutionalized weight‐reducing programme combined with a dietary plan and aerobic exercise. This therapeutic approach lead to loss of fat mass in both sarcopenic obese and non‐sarcopenic obese women, and it lead to an improvement in the lipid‐lipoprotein profile. However, lean mass index differences between the two groups remained significant even at the end of the weight‐reducing programme. The authors concluded that such treatment approaches may have deleterious effects on lean mass only in women who are non‐sarcopenic obese.^29^ Creatine supplementation has been used by Pinto *et al*.^30^ as part of a 12‐week double‐blind, randomized, placebo‐controlled trial in elderly subjects. Creatine supplementation plus resistance training was shown to have superior gains in lean mass when compared with the placebo group that also underwent resistance exercise training. However, this was not associated with an increase in the 10 repetition maximal tests in bench press and leg press exercise, which buttresses the view that increases in lean mass are not always associated with increases in strength, and ideal medication should combine an increase in both.^30^ Finally, a battle of ongoing debate remains the questions whether or not certain drugs might be associated with increases in muscle loss. Such discussion is, for example, ongoing with regard of using statins in elderly or terminally ill patients.^31^

### Animal data

As outlined previously, changes in skeletal muscle structure and function are found in various clinical populations. However, the degradation of muscle fibres remains incompletely understood. The ubiquitin‐proteasome system plays a pivotal role in this context. The muscle specific RING‐finger (MuRF) protein family of E3 ligases is of particular importance in this context. Lodka *et al*. produced MuRF2 and MuRF3 double knock‐out mice and investigated skeletal muscle and heart by morphological measurements, histological analyses, electron microscopy, immunoblotting, and real‐time polymerase chain reaction. The authors found that affected mice showed a protein aggregate myopathy in skeletal muscle with reduced maximum force development and concluded that the redundant function of MuRF2 and MuRF3 is important for the maintenance of skeletal muscle and cardiac structure and function *in vivo*.^32^ Data by Nederveen *et al*. showed that a greater distance exists between capillaries and type II fibre‐associated skeletal muscle satellite cells suggesting implications in the adaptation of skeletal muscle to exercise.^33^ Likewise, mice with sarcopenia appear to have a marked defect of autophagy. Autophagy is part of the normal maintenance of muscle mass and function.^34^ Cationic influx into skeletal muscle via degenerine channel activation appears to cause caspase degradation of cytosolic proteins, which has been viewed as a result of mitochondrial dysfunction in ageing and sarcopenia.^35^

On a treatment level, recent data suggest that short‐term supplementation of docosahexaenoic acid may help in increasing energy stores and therefore in preserving muscle mass in response to fasting.^36^ Dihydrotestosterone treatment, on the other hand, was able to rescue the decline in protein synthesis in isolated skeletal muscle fibres from the mouse.^37^ The latter finding is important, because testosterone supplementation has been used in several clinical settings as part of smaller studies to preserve muscle mass and to increase functional capacity. Apart from the ubiquitin‐proteasome system and autophagy, the caspase system particularly involving apoptosis using Caspase‐3 seems to be another important trigger for age‐related muscle loss.^38^ Kob *et al*. found that male rats were more prone to the decline of muscle during ageing than female animals. This decline was further enhanced by long‐term high‐fat diet.^39^ Previous studies have reported that caloric restriction leads to an increase in the lifespan of rodents and might be able to reduce age‐induced muscle loss. Van Norren *et al*.^40^ performed a study in mice that received either an *ad libitum* diet (control) or a diet matching 70% of the intake of the control group. The authors then measured daily activity, body composition, grip strength, insulin sensitivity, and general agility and balance. Caloric‐restricted animals were found to have only half the weight of the control animals from the age of 12 months onwards. Interestingly, the control group showed a decline in hind limb muscle mass starting at the age of 24 or 28 months, which was not present in the caloric‐restriction group, and the study confirms that caloric‐restriction animals present with less sarcopenia prevalence than those eating *ad libitum*. The authors concluded that the positive effect of caloric restriction of muscle maintenance may not be only a direct consequence of lower energy intake but may also be related to more active behaviour in a specific time frame.^40^ Mogi *et al*. found that diabetes may enhance sarcopenic obesity development for a mechanism involving an anomalous fibro‐adipocyte progenitor cell differentiation.^41^ However, even though single studies can help us to understand the loss of skeletal muscle during the ageing process or during disease states, the list of mediating factors is very long including, for example, expression of protocadherin gamma.^42^ Other studies have shown beneficial effects on skeletal muscle mass or muscle performance using, for example, the metabolic modulator trimetazidine^43^ or the melanocortin receptor type 3 agonist d‐Trp(8)‐γMSH that decreases inflammation and muscle wasting in rats with arthritis.^44^

## Heart failure

Cardiac cachexia as a consequence of advancing heart failure has been described as an independent risk factor of death already in 1997.^45^ Sarcopenia as a consequence of heart failure has only been described recently, and it was shown that patients with muscle wasting fulfilling the criteria of sarcopenia and heart failure have significantly reduced muscle strength and exercise capacity.^46^, ^47^ In patients with heart failure, muscle wasting can be approached by aerobic exercise training,^48^ which is being advocated for these patients irrespective of wasting being present or not as long as patients are clinically stable.^49^ Several other factors have been highlighted recently in the setting of heart failure. Using data from a rat model of heart failure developed after ligation of the left anterior decending coronary artery, it was shown that divergent antioxidant and metabolic responses exist in the diaphragm, and the quadriceps muscle with glutathione peroxidase and manganese superoxide dismutase activity increased in the diaphragm but reduced in the quadriceps.^50^ Metabolic enzymes were unaltered in the diaphragm, but cytochrome c oxidase activity decreased, and lactate dehydrogenase activity increased in the quadriceps of animals with heart failure. Catabolic responses remained similar between heart failure and non‐heart failure animals. In summary, MuRF‐1 and proteasome activity were elevated, and oxidative enzyme activity failed to increase in the diaphragm of animals with heart failure. This likely suggests that a myopathy is present in respiratory muscles of heart failure animals, which is interesting to note because this muscle remains in constant use.^50^, ^51^ In addition, adiponectin resistance has been recently reported in skeletal muscle of patients with chronic heart failure. Sente *et al*. proposed that the resistance process may be multifactorial and may lead to an integration of abnormalities from insulin signalling, mitochondrial biogenesis, and ceramide metabolism.^52^ Similarly, other factors have been involved in the development of skeletal muscle loss in patients with heart failure. In particular, the expression of skeletal muscle fibronectin type III domain containing 5 gene in patients with ischaemic cardiomyopathy as it appears that the reduced expression is modulated be inflammatory cytokines and angiotensin‐II. This factor may be involved in the slowing of browning of adipocytes.^53^ Rozentryt *et al*. showed that serum phosphorus is associated with a catabolic/anabolic imbalance in heart failure using data from 1029 stable patients of heart failure. These authors found that a catabolic/anabolic imbalance, N‐terminal pro‐B‐type natriuretic peptide values, serum sodium, kidney function, age, and sex were independent predictors of serum phosphorus. Thus, the authors concluded that metabolic status is an independent determinant of serum phosphorus in heart failure.^54^

## Chronic obstructive pulmonary disease and mechanical ventilation

Cachexia and muscle wasting are common in the course of COPD. Both are recognized to have strong impact on disease progression and patients' prognosis.^55^ Recent evidence suggests that the expression of growth differentiation factor‐15 (GDF‐15) may be involved in the loss of skeletal muscle mass in patients with COPD. Patel *et al*.^56^ assessed the association of GDF‐15 expression with muscle mass and exercise performance and found increased serum values as well as increased expression in skeletal muscle in patients with COPD compared with controls. In particular, circulating GDF‐15 was inversely correlated with rectus femoris cross‐sectional area and exercise capacity. However, no association was found with body mass index. The authors therefore assume that GDF‐15 contributes to loss of muscle mass in patients with COPD.^56^ Lewis *et al*.^57^ identified microRNAs associated with low fat‐free mass phenotype in patients with COPD using a polymerase chain reaction screen of 750 microRNAs. They found that imprinted miR‐675 and a cluster including miR‐519a were differentially expressed in the quadriceps of patients with low fat‐free mass index compared with those with normal values. In particular, the association of miR‐519a expression with fat‐free mass index was present in male patients with severe COPD, and *in vitro* miR‐675 inhibited myoblast proliferation. The authors therefore concluded that increased expression of miR‐675 and also altered methylation of the H19 imprinting control region are associated with a low fat‐free mass index in patients with COPD.^57^ Just like in patients with COPD, muscle weakness and muscle wasting are frequently observed in critically ill patients on the intensive care unit. These patients are at particular risk because such muscle wasting may be involved in the failure of weaning from mechanical ventilation.^58^

## Cancer

### Pathophysiology

Cachexia development in patients with cancer is of a multi‐dimension nature. The prevalence varies strongly according to tumour type between 28% in patients with colorectal cancer and 57% or more in patients with head and neck or pancreatic cancer.^59^, ^60^ Yet still cachexia remains under recognized.^61^ The experience of weight loss differs between patients and care givers.^62^ The multi‐dimensional aspect of wasting in patients with cancer is buttressed by the fact that anorexia is commonly present in patients with cancer and a major component of cancer cachexia. Anorexia in itself has multiple causes such as substance‐release from the tumour (e.g. pro‐inflammatory cytokines), dysphagia caused by the tumour itself, the alteration of nutrients by the tumour (e.g. zinc deficiency), tumours causing tissue hypoxia, increased accumulation of serotonin by increased peripheral tryptophan amounts, or the alterations of the release of peripheral hormones that alter feeding.^63^ Even though an understanding of anorexia pathophysiology is being developed, therapeutic approaches in this regard remain unsatisfying. Different drugs such as megestrole acetate, cannabinoids, and ghrelin agonists have been used with mixed effectiveness at best. Another phenomenon commonly encountered in patients with cachexia is hypermetabolism. Dev *et al*. studied 60 patients with advanced cancer and weight loss exceeding 5% or anorexia who underwent indirect calorimetry to measure resting energy expenditure. Most patients had a solid tumour, predominately gastrointestinal cancers. Hypermetabolism was present in 58% of the patients in the study, buttressing the view that this phenomenon is very frequent in patients with cancer.^64^ However, it remains unclear whether hypermetabolism might be an appealing therapeutic target.^65^ Moryoussef *et al*. studied 31 patients with advanced or high‐risk resected gastrointestinal stroma tumours and found that 12 of these patients were sarcopenic. Approximately 63.6% of the assessable sarcopenic patients became non‐sarcopenic after 6 months of imatinib therapy, and the authors therefore conclude that sarcopenia might be reversible in some patients with gastrointestinal stroma tumours who were treated with imatinib.^66^

### Prognosis

Several biomarkers have been used in body wasting to predict outcomes and survival. Recent evidence shows that GDF‐15 levels, a marker that has been used several times,^67^ were increased in patients with cancer‐related weight loss just like interleukin‐6 and interleukin‐8.^68^ This was also true for interleukin‐1 receptor antagonist (IL‐1RA), interleukin‐4, interferon‐γ, and tumour necrosis factor. Patients with cancer‐related weight loss had lower handgrip strength, lower appendicular lean body mass, and fat mass as well as a lower Eastern Cooperative Oncology Group and Karnofsky performance scores. Lerner *et al*. found that GDF‐15, interleukin‐6, and interleukin‐8 correlated significantly with weight loss, and in particular, GDF‐15 was negatively correlated with appendicular lean body mass, hand grip strength, and fat mass. The authors suggest that GDF‐15 may serve as a prognostic indicator in patients with cancer.^68^ Stephens *et al*. studied 107 subjects, 92 of whom had upper gastrointestinal cancer, and the remaining were controls. Rectus abdominis muscle specimens were obtained at surgery and analysed with regard to the expression of Akt and phosphorylated Akt as well as forkhead box O (FOXO) transcription factors, ubiquitin E3 ligases, BIP3, and GABARAPL1 as well as other markers, particularly myosin heavy chain and dystrophin. The latter two markers were able to predict survival in patients with lower levels of myosin heavy chain and dystrophin.^69^ As discussed earlier, there is poor sensitivity and specificity for biomarkers in any type of wasting whether cachexia or sarcopenia. Nyasavajjala *et al*.^70^ evaluated creatinine and myoglobin as predictors of anaerobic threshold in patients with colorectal cancer. For this purpose, they recruited 47 patients with colorectal cancer and a matching number of healthy volunteers and assessed their body composition using DEXA scan and their exercise capacity using the measurement of anaerobic threshold on spiroergometry. They found that in spite of differences in the aerobic capacity, no differences were found with regard to the serum levels of myoglobin and creatinine between the two groups. However, anaerobic threshold was significantly lower in the colorectal cancer group as compared with the control group, and there was a significant correlation between lean muscle mass and anaerobic threshold. No such correlation was found with regard to myoglobin and creatinine, and the authors therefore conclude that their predicted value remains low.^70^

### Treatment

**Figure 2:**
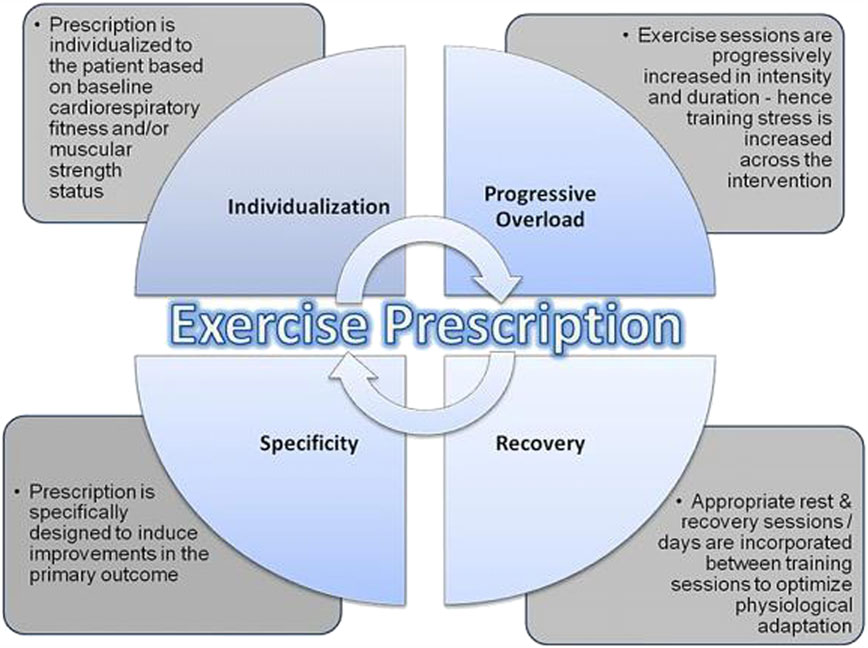
The principles of training. Reproduced from Sasso *et al*. 72

Exercise training has been advocated in recent years as having positive effects on exercise capacity and overall well‐being, quality of life, and potentially even of prognosis. Exercise training is one of the mainstays in the maintenance of muscle mass in patients with cachexia or sarcopenia as long as such approach is possible from a physical point of view for the patient. A recent Cochrane Collaboration systematic review showed disappointing results after screening more than 3000 separate manuscripts, because most authors who did explore the concept of exercise training did so only in patients with cancer but not in cancer cachexia.^71^ Still, a small number of intervention studies have investigated the impact of exercise training in patients with cancer cachexia. Sasso *et al*.^72^ have provided intriguing insight and suggest a framework for the prescription of exercise in patients with cancer (*Figure* 2). They concluded that the vast majority of studies have tested the efficacy of an exercise prescription that adhered to traditional guidelines consisting of either supervised or home‐based endurance training or endurance training combined with resistance training. The prescription in these studies that did not target cachexia patients but rather oncology patients *per se* was typically prescribed at a moderate intensity of 50–75% of predetermined physiological parameters, typically age‐predicted heart rate maximum or reserve. The usual prescription frequency was 2–3 sessions per week of 10‐ to 60‐min duration, and the usual time frame was 12–15 weeks.^72^

As mentioned before, there are, at present, no therapeutic interventions being established for the treatment of muscle wasting and sarcopenia or cachexia. However, exercise training is known to exert anti‐inflammatory and anti‐oxidative effects that are able to attenuate signalling pathways associated with protein degradation, which renders this therapeutic concept very interesting.^73^ Such pathways include, for example, muscle regulatory factors, which were studied by Slimani *et al*.^74^ using a tibialis anterior muscle atrophy model in rats that were subjected to hindlimb cast immobilization for 8 days. They found that remobilization correlated with reduced fibre cross‐sectional area and thickening of the endomysium. Integrin‐linked kinase protein levels increased during immobilization and remobilization after 10 days, and this was inversely correlated with changes in Akt phosphorylation. The authors concluded that tibialis anterior muscle atrophy can be counterbalanced during remobilization only when the terminal differentiation step of regeneration is enhanced.^74^ Indeed, the number of pathways and players involved in the development of muscle atrophy and sarcopenia is increasing steeply, and it is sometimes difficult to keep track. Marino *et al*.^75^ studied increased levels of atrogin‐1, MuRF‐1, Beclin‐1 p62, Smad‐2, and activin‐A in a mouse model of muscle wasting. Overexpression of activin‐βC antagonized the ubiquitin‐proteasome system activity and the autophagic‐lysosomal degradation pathways yielding reduction in the serum levels of activin‐A. The authors therefore suggest that activin‐βC may be a novel therapy to abrogate cancer‐associated weight loss and potentially even prolonging survival.^75^

From a clinical standpoint, nutritional approaches as well as pharmacological interventions remain important.^76^ Stewart Coats *et al*.^77^ studied 87 patients with stage III or IV colorectal cancer or non‐small cell lung cancer who were cachetic and received one of two doses of espindolol or placebo. Espindolol is a novel non‐selective beta‐blocker with central 5‐HT1a and partial β_2_ receptor agonist effects. After 16 weeks of treatment, patients on high‐dose espindolol showed a significant increase in lean body mass and handgrip strength. Stair climbing power and 6‐min walk distance remained statistically non‐significant but showed an effect favouring high‐dose espindolol. Overall, espindolol was well‐tolerated, and the authors suggest to study the drug in a larger cohort of patients.^77^, ^78^ Besides exercise capacity, fatigue is an important problem affecting patients under palliative care. A recent Cochrane Collaboration systematic review screened more than 1600 publications using data from 18 drugs and almost 4700 participants. Unfortunately, the authors concluded that because of the heterogeneity of the trials, they could not recommend a specific drug for the treatment of fatigue in palliative care patients. However, the authors highlighted amantadine, methylphenidate, and modafinil to be further researched in larger settings.^79^ A similar standpoint has been advocated for anamorelin.^80^ On the other hand, a lack of regulatory guidance has been deplored, as it is not entirely clear what kind of endpoints in clinical trials of cachexia will be acceptable for regulatory bodies such as the Food and Drug Administration or the European Medicines Agency.^81^

From a therapeutic perspective, some studies also tested the effect of chemotherapy regimens on muscle wasting. Rutten *et al*.^82^ found that patients with ovarian cancer have worst survival when they loose skeletal muscle during neoadjuvant chemotherapy. However, their study remained small with only 123 ovarian cancer patients treated with neoadjuvant chemotherapy and interval debulking in the Netherlands. The proteasome inhibitor bortezomib was studied only in an animal model of muscle wasting in cancer cachexia that was induced in rats by i.p. injection of a hepatoma cell line.^83^ Even though bortezomib administration reduced proteasome and nuclear factor‐κB activity in skeletal muscle after tumour transplantation, this effect did not prevent body weight loss and muscle wasting. The authors concluded that further studies are needed to address this issue. Toledo *et al*. studied a multifactorial treatment design including formoterol and megestrol acetate in cachectic tumour‐bearing rats undergoing chemotherapy with sorafenib. Sorafenib is known to decrease tumour cell content; however, cachexia occurrence in affected animals persists. The authors found that the combination of formoterol and megestrol acetate may be a promising strategy for treating cancer cachexia in a preclinical setting and suggest that a clinical trial involving cachectic cancer patients may be warranted.^84^ Another study investigated the effects of megestrol acetate in improving cardiac function in a model of cancer cachexia‐induced cardiomyopathy by autophagic modulation.^85^ The authors found that the use of megestrol acetate, an appetite stimulating, improves the survival and reduces wasting through a marked down‐regulation of autophagy, both in skeletal and cardiac muscle. Particularly, the latter effect leads to a significant improvement in cardiac function.^85^ Chen *et al*.^86^ found that ghrelin prevents tumour‐induced and cisplatin‐induced muscle wasting. They demonstrated that implantation of a Lewis lung carcinoma cell line into adult male mice together with the use of cisplatin‐induced muscle atrophy by activating pro‐inflammatory cytokines, myostatin, and p38‐C/EBP‐β and by down‐regulating Akt, myoD, and myogenin altogether leading to an activation of the ubiquitin‐proteasome system. Ghrelin was able to prevent these changes *in vivo* and *in vitro* by significantly increasing muscle mass and grip strength as well as by improving survival. In this context, it is interesting to note that the diet composition may be a source of variation in animal models of cancer cachexia. Giles *et al*.^87^ recommend that minimum standards should be used for diet definition in models of cachexia, which includes the reporting of diet content and composition as well as food intake to improve reproducibility of preclinical studies. Likewise, Dwarkasing *et al*.^88^ found that altered serotonin signalling is associated with changes in food intake behaviour in cachectic mice and suggest that serotonin regulation might be a therapeutic target to prevent the development of cancer‐induced eating disorders. Because essential amino acids are indeed an essential component of muscle structure, particularly the branched‐chain amino acids valine, isoleucine, and leucine, a study by Toneto *et al*.^89^ put a particular focus on nutritional leucine supplementation. They found that a leucine‐rich diet could modulate heart damage, cardiomyocyte proteolysis, and apoptosis driven by cancer cachexia in a model of male rats that were inoculated with a subcutaneous Walker‐256 carcinoma cell line. All in all, it appears that nutrition screening is important as well as nutritional supplements in the treatment of patients with cachexia—particularly in those with cancer.^90^ Having said this, the importance of also screening for severe dysphagia cannot be stressed enough. Wakabayashi *et al*. found skeletal muscle to be associated with severe dysphagia in patients with cancer.^91^, ^92^ However, it remains important that not only patients are affected by eating disorders or eating‐related distress but this can also involve bereaved family members. Amano *et al*.^93^ performed a study in 133 inpatient hospices in Japan involving a total of 702 bereaved family members and found that family members of advanced cancer patients may experience high levels of eating‐related distress and may require nutritional counselling.

## Conclusions

The aforementioned text describes advances in the field of cachexia, sarcopenia, and wasting research. It is reassuring that reasonable progress is being made; however, it is also quite disappointing that this progress is made at such low speed. We have a huge number of biomarker candidates for the diagnosis and prognosis of muscle wasting, but none is ready for clinical use. We have numerous candidate drugs, but none is ready for clinical use unless one may count megestrol acetate that has approval only in the setting of breast cancer. The aforementioned text also describes one of the major problems that we are facing in cachexia and wasting research: the lack of randomized controlled trials of reasonable size. Therefore, we are—for the time being—left with nutritional counselling and the use of exercise training. Some hope remains that the *Journal of Cachexia, Sarcopenia and Muscle* will soon report novel insight into pathophysiology and—most importantly—treatments of wasting disorders.

## Conflict of interest

None declared.
